# Repeated Bout Effect Was More Expressed in Young Adult Males Than in Elderly Males and Boys

**DOI:** 10.1155/2013/218970

**Published:** 2013-01-01

**Authors:** Giedrius Gorianovas, Albertas Skurvydas, Vytautas Streckis, Marius Brazaitis, Sigitas Kamandulis, Malachy P. McHugh

**Affiliations:** ^1^Department of Applied Biology and Physiotherapy, Research Centre for Fundamental and Clinical Movement Sciences, Lithuanian Sports University, Sporto 6, 4422 Kaunas, Lithuania; ^2^Nicholas Institute of Sports Medicine and Athletic Trauma, Lenox Hill Hospital, 130 East 77th Street, New York, NY 10075, USA

## Abstract

This study investigated possible differences using the same stretch-shortening exercise (SSE) protocol on generally accepted monitoring markers (dependent variables: changes in creatine kinase, muscle soreness, and voluntary and electrically evoked torque) in males across three lifespan stages (childhood versus adulthood versus old age). The protocol consisted of 100 intermittent (30 s interval between jumps) drop jumps to determine the repeated bout effect (RBE) (first and second bouts performed at a 2-week interval). The results showed that indirect symptoms of exercise-induced muscle damage after SSE were more expressed in adult males than in boys and elderly males, suggesting that the muscles of boys and elderly males are more resistant to exercise-induced damage than those of adult males. RBE was more pronounced in adult males than in boys and elderly males, suggesting that the muscles of boys and elderly males are less adaptive to exercise-induced muscle damage than those of adult males.

## 1. Introduction

Although a large amount of research relates to exercise-induced muscle damage in adult subjects [1–7], very little research has assessed the differences in fatigue between children, adults, and elderly persons when doing the same exercise protocol—one that causes exercise-induced muscle damage [8–13]. In addition, there is a lack of research regarding the effect of age on repeated bout effect (RBE) [11, 12]. We found no study in which the effects of age (boys versus adults versus elderly) on RBE were assessed using the same exercise protocol. Also, we did not come across any special studies devoted to analysis of the effect of age on various indirect symptoms of muscle damage (i.e., creatine kinase (CK) levels, muscle soreness, and especially both voluntary (including muscle voluntary activation index) and electrically induced muscle force at different frequencies after exercise that cause mechanical muscle damage). Therefore, the effect of the same exercise-induced muscle damage protocol on RBE indicators in males across three lifespan stages (i.e., childhood, adulthood, and old age) remains unclear, especially as regards accepted monitoring markers (i.e., CK, muscle soreness, decrease in electrically induced torque, and maximal voluntary contraction force of quadriceps muscle (MVC)). However, it has been well documented that the muscles of adults contain relatively more fast twitch muscle fibers (type II) than do the muscles of children and elderly males [14–16]; therefore, we hypothesize that muscles of children and elderly males might be more resistant to exercise-induced muscle damage than those of adult males and that RBE might be more expressed in subjects whose muscles are more sensitive to muscle damage, that is, in young adult males than in boys and elderly males. Therefore, the main purpose of this study was to test the hypothesis.

## 2. Materials and Methods

### 2.1. Participants

Healthy untrained boys (age 11.8 ± 0.9 years, body mass 40.2 ± 7.9 kg, height 149.3 ± 8.3 cm, and body mass index (BMI) 17.7 ± 1.6, *n* = 11), young adults (age 20.8 ± 1.9 years, body mass 82.6 ± 10.9 kg, height 181.9 ± 5.9, BMI 25.0 ± 2.6, *n* = 11), and elderly males (age 63.2 ± 3.6 years, body mass 78.4 ± 13.9 kg, height 176.0 ± 6.5, BMI 23.4 ± 3.9, *n* = 11) gave their informed consent to take part in the experiment within the study. The subjects were physically active but did not take part in any formal physical exercise or sport program. Each subject read and signed written informed consent form consistent with the principles outlined in the Declaration of Helsinki. This study was approved by the Ethics Committee of Kaunas University of Medicine.

### 2.2. Experimental Protocol

Three to five days before the experiment, subjects were familiarized with electrical stimulation and different tasks of voluntary performance. On the day of the experiment, after measurement of CK in the blood, the subjects completed warm-up exercises that consisted of 5 min of running on the spot at an intensity that corresponded to heart rates of 110–130 beats·min^−1^. This was followed by 10 squat-stands. After their warm-up, a subject was seated in the experimental chair and after 5 min muscle contractile properties were recorded in the following sequence: P20, P100, and MVC. Quadriceps muscle voluntary activation index was registered during MVC. About 3 min later, the SSE was undertaken. Two to five min and 48 h after SSE, recordings were made of the same contractile properties, both voluntary and electrostimulation-evoked muscle contraction properties. At 24 h and 48 h after SSE, muscle soreness and CK activity were determined. The experimental protocol was performed twice, first under control conditions (*bout* 1) and then 2 weeks later (*bout* 2).

### 2.3. Muscle Damaging Stretch-Shortening Exercise (SSE)

The subjects performed 100 intermittent (30 s interval between the jumps) drop jumps (DJs) from the height of 0.5 m with countermovement to 90 ± 3 degrees angle in the knee and immediate maximal rebound. During the jumps hands of the subjects were on the waist. The subject stepped on 0.5 m high platform with his left leg, that is, the leg in which muscle contraction force was not tested. After each jump the subjects were informed of the height and knee joint angle of the jump and were motivated to perform each jump as high as possible. Knee joint angle during drop jumps was controlled using electronic goniometer (Biometrics Ltd. Model SG150, UK) connected to Angle Display Unit device (Biometrics Ltd. Model ADU301, UK). Before SSE and during recovery control drop jumps were performed with the same techniques as during SSE. Height of the DJ was calculated by an earlier technique applying the following formula: *h* (cm) = *g* × *t* ^2^/8, where *h* = height of the drop jump, *g* = acceleration of gravity (9.81 m × s^−2^), and *t* = flight time (s) [17]. SSE was performed making use of the multicomponent Kistler force plate (type 9286A, USA). A similar research protocol was applied in previous research [6, 18, 19].

### 2.4. Isometric Torque and Electrical Stimulation

The isometric torque of knee extensor muscles was measured using an isokinetic dynamometer (System 3; Biodex Medical Systems, Shimley, New York). The sensitivity of the Biodex System 3 in torque measurements is ±1.36 Nm. The subjects sat upright in the dynamometer chair with the knee joint positioned at 120 degrees angle (180 degrees—knee full extension). The equipment and procedure for electrical stimulation were essentially the same as previously described [4–6]. Direct muscle stimulation was applied using two carbonized rubber electrodes, covered with a thin layer of electrode gel (ECG-EEG Gel; Medigel, Modi'in, Israel). One of the electrodes (6 × 11 cm) was placed transversely across the width of the proximal portion of the quadriceps femoris. Another electrode (6 × 20 cm) covered the distal portion of the muscle above the patella. A standard electrical stimulator (MG 440; Medicor, Budapest, Hungary) was used. The electrical stimulation was delivered in square-wave pulses, 0.5 ms in duration. The tolerance of volunteers to electrical stimulation was assessed on a separate occasion. All participants showed good compliance with the procedure and were recruited for the study. The intensity of electrical stimulation was selected individually by applying single stimuli to the muscles tested. During this procedure, the current was increased until no increment in single twitch torque could be detected by an additional 10% increase in current strength. The output from the force transducer was also displayed on a voltmeter in front of the subject.

The following data were measured: the torque at 20 Hz (P20) and 100 Hz (P100) of electrical stimulation using 1 strains of stimuli at each frequency, respectively. MVC at the knee angles of 120 degrees, peak of the MVC was, reached and maintained some 5 seconds before relaxation, twice. The rest interval between muscle electrostimulations was 10 s and between MVC measurements it was 2 min. The change in the ratio of P20/P100 after exercise was used for the evaluation of low frequency fatigue (LFF) [4, 7, 20].

### 2.5. Voluntary Activation Index (VA) Measurement

The volunteer was positioned in the dynamometer chair, and the stimulating electrodes were placed on the right leg. After a 5 min rest, two 5 s MVC readings separated by a 2 min rest interval were obtained. After ~3 s of MVC, a 250 ms test train of stimuli at 100 Hz (TT-100 Hz) was superimposed on the voluntary contraction. The TT-100 Hz was repeated 1-2 s after the MVC. TT-100 Hz contractions were used to assess voluntary activation of knee extensors. The amplitude of the superimposed tetani was calculated relative to the baseline, which was defined as the average torque over a period of 1 s immediately before stimulation. The superimposed TT-100 Hz produced measurable torque increments in all subjects. For the VA, the TT-100 Hz torque of the relaxed muscles was used as the control torque, and the following formula was applied: VA (%) = {1 − (superimposed TT-100 Hz torque/control TT-100 Hz torque)} × 100 per cent [4, 19, 21].

### 2.6. Plasma Creatine Kinase (CK) Activity

Approximately 5 mL of blood was drawn from *vena cubiti media* of the arm at each measurement time point (before exercise as well as 24 h and 48 h after exercise). Plasma samples were pipetted into microcentrifuge tubes and stored in a −20°C freezer until analysis. Plasma creatine kinase activity (IU/L) was determined by using automatic biochemical analyzer “Monarch” (Instrumentation Laboratory SpA, USA-Italy). The normal reference range for men for CK using this method is between 24 and 195 IU·L^−1^ according to the manual provided with the analyzer.

### 2.7. Muscle Soreness

Muscle soreness was reported subjectively using a visual analogue scale from 0 to 10 points. Each number on the scale has descriptive words for soreness: 0 (none), 1 (very slight), 2 (slight), 3 (mild), 4 (less than moderate), 5 (moderate), 6 (more than moderate), 7 (intense), 8 (very intense), 9 (barely tolerable), and 10 (intolerably intense). The participants were required to indicate the severity of soreness in their quadriceps during 2-3 squats at 24 and 48 h after *bouts* 1 and 2 [4–6].

### 2.8. Statistical Analyses

The Kolmogorov-Smirnov test confirmed that all data were normally distributed. Three-way analysis of variance (ANOVA) for repeated measures was used to determine the effect of age (three groups), time (before, 2–5 min after and 48 h after exercise), and RBE (*bout* 1 and *bout *2) on the variables. Significant differences were subjected to post hoc testing using paired *t*-tests with Bonferroni correction for multiple comparisons. Descriptive data are presented as the mean ± SD. The level of significance was set at 0.05. The statistical powers (SP) for all mechanical indicators were calculated using an alpha level of 0.05, a sample size of 11, the standard deviation, and the average value before and after SSE. 

## 3. Results

### 3.1. Stretch-Shortening Exercise

**Figure 1:**
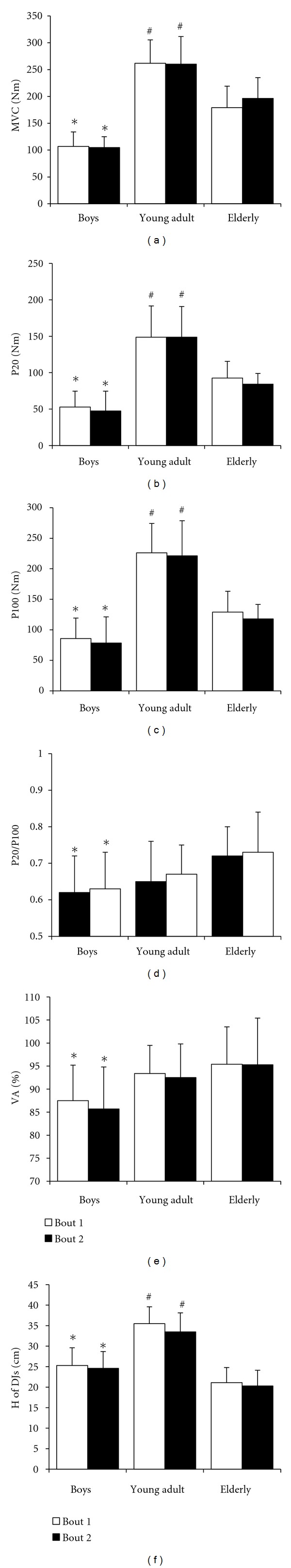
Voluntary (MVC (a) maximal voluntary contraction force of quadriceps muscle; *H* of DJs (f) height of drop jumps; VA (e) quadriceps muscle voluntary activation index) and electrically (P20 (b) and P100 (c) muscle contraction force evoked by stimulating quadriceps muscle at 20 Hz and 100 Hz frequencies, resp.; P20/P100 (d) ratio of P20/P100 indicates the level of low frequency fatigue) induced muscle performance before *bout* 1 and *bout* 2 in boys (*n* = 11), young adult males (*n* = 11), and elderly males (*n* = 11) (mean ± SD). *P < 0.01, between elderly males and boys; ^#^ *P* < 0.001, compared with young adult males and boys.

The control (before SSE) *H* of DJs was significantly greater in young adults than in boys and elderly males (*P* < 0.001; SP > 80%); however, in boys it was significantly greater than in elderly males (*P* < 0.05; SP = 45.4%) (Figure 1). Average *H* of DJs during 100 DJs in boys, young adult males, and elderly males was 23.4 ± 3.4 cm, 31.9 ± 4.2 cm, and 19.1 ± 3.3 cm, respectively, during *bout* 1 (*P* < 0.01, compared between groups, SP > 80%), and 24.3 ± 3.9 cm, 32.5 ± 4.2 cm, and 19.9 ± 1.5 cm during *bout* 2 (*P* < 0.01, compared between groups, SP > 80%; *P* > 0.05 compared between *bout* 1 and *bout* 2, SP > 30%). The average contact time of 100 DJs in boys, young adult males, and elderly males was 0.58 ± 0.06 s, 0.63 ± 0.09 s and 0.86 ± 0.09 s, respectively, during *bout* 1 (*P* < 0.05, compared between groups, SP > 30%), and 0.57 ± 0.06 s, 0.64 ± 0.09 s, and 0.82 ± 0.1 during *bout* 2 (*P* < 0.05, compared between groups, SP > 30%; *P* > 0.05 compared between *bout* 1 and *bout *2, SP > 35%). Thus, there was no significant difference in SSE loads between *bout* 1 and *bout* 2 in all groups.

### 3.2. Voluntary and Electrically Induced Muscle Performance before *bout* 1 and *bout* 2

**Figure 2:**
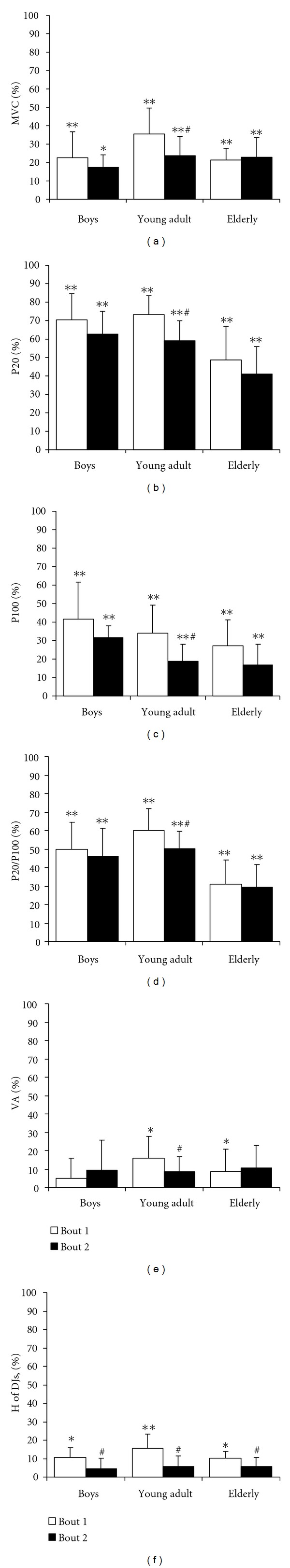
Voluntary (MVC (a) maximal voluntary contraction force of quadriceps muscle; *H* of DJs (f) height of drop jumps; VA (e) quadriceps muscle voluntary activation index) and electrically (P20 (b) and P100 (c) muscle contraction force evoked by stimulating quadriceps muscle at 20 Hz and 100 Hz frequencies, resp.; P20/P100 (d) ratio of P20/P100 indicates the level of low frequency fatigue) induced muscle performance after *bout* 1 and *bout* 2 in boys (*n* = 11), young adult males (*n* = 11), and elderly males (*n* = 11) (mean ± SD). ^∗,∗∗^ *P* < 0.05 and *P* < 0.001, compared with the before-exercise level; ^#^ *P* < 0.05, compared with *bout* 1.

There were no significant differences for all registered parameters before *bout* 1 and *bout* 2 in all groups (Figure 2). Preexercise electrically induced (P20 and P100) and voluntary (MVC) knee torques were significantly greater in young adults than in elderly males and boys (*P* < 0.001; SP > 80%); however, in elderly males they were significantly greater than in boys (*P* < 0.01) (Table 1). The VA index and P20/P100 of boys were significantly lower than in elderly males (*P* < 0.01; SP = 35.4%).

### 3.3. Differences in RBE between Boys, Young Adult Males, and Elderly Males: Immediately after SSE

RBE manifested immediately after SSE in all voluntary and electrically induced muscle performance in young adult males, while in boys and elderly males RBE was evident only in *H* of DJs (Figure 2). Also, the contact time of DJs did not change significantly after SSE in all groups. Electrically induced muscle force P20 (*P* < 0.001; SP > 80%), P100 (*P* < 0.001; SP > 80%), P20/P100 (*P* < 0.001; SP > 80%), and MVC (*P* < 0.001; SP > 80%) decreased significantly in all groups after *bout* 1 and *bout* 2, whereas *H* of DJs decreased significantly (*P* < 0.05; SP = 49.5%) after *bout* 1 in all groups and VA (*P* < 0.05) only in young adults after *bout* 1. It is of interest that changes in P20/P100 were significantly less in elderly males compared with young adults and boys (*P* < 0.05). Thus, RBE in changes of voluntary and electrically induced muscle force immediately after SSE was more pronounced in young adults.

### 3.4. Differences in RBE between Boys, Young Adult Males, and Elderly Males: 48 h after SSE

**Figure 3:**
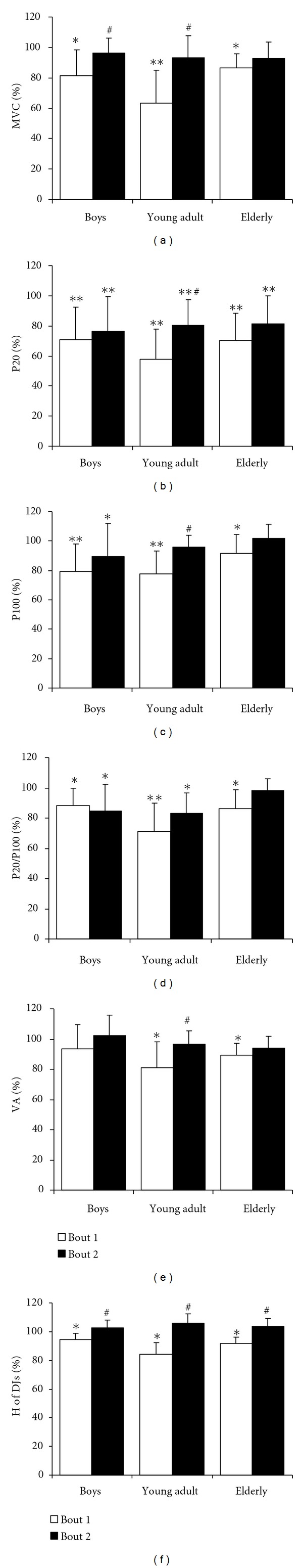
Voluntary (MVC (a) maximal voluntary contraction force of quadriceps muscle; *H* of DJs (f) height of drop jumps; VA (e) quadriceps muscle voluntary activation index) and electrically (P20 (b) and P100 (c) muscle contraction force evoked by stimulating quadriceps muscle at 20 Hz and 100 Hz frequencies, resp.; P20/P100 (d) ratio of P20/P100 indicates the level of low frequency fatigue) induced muscle performance 48 h (percent from before) after *bout* 1 and *bout* 2 in boys (*n* = 11), young adult males (*n* = 11), and elderly males (*n* = 11) (mean ± SD). ^∗,∗∗^ *P* < 0.05 and *P* < 0.001, compared with before; ^#^ *P* < 0.05, compared with *bout* 1.

Electrically (P20, P100, and P20/P100) and voluntary induced (MVC, *H* of DJs) muscle performance did not recover within 48 h after *bout* 1 in all groups (Figure 3). Voluntarily muscle performance (MVC and *H* of DJs) fully recovered within 48 h after *bout* 2 in all groups. It is of interest that VA did not recover to the initial level within 48 h after *bout* 1 in young adult and elderly males. 

### 3.5. Changes in CK and Muscle Soreness

The CK activity in the blood increased significantly in all groups 24 and 48 h after SSE (*P* < 0.05; SP > 80%) (Table 1). However, CK activity in young adult males 24 h and 48 h after SSE was significantly greater than in boys and elderly males (*P* < 0.001; SP > 80%). There was no significant difference in CK 24–48 h after *bout* 1 and *bout* 2 between boys and elderly males. Differences in CK 24 h in boys, young adult males and elderly males between *bout* 1 and *bout* 2 were 240.5 ± 188.8, 587.8 ± 568.5, and 437.0 ± 110.2 IU/L, respectively (*P* < 0.01 between groups; SP > 80%). Muscle soreness in boys, young adult males and elderly males was 5.7 ± 1.8, 6.2 ± 2.1, and 3.7 ± 1.1 points, respectively, 24 h after *bout* 1 and 4.9 ± 2.8, 6.3 ± 2.1, and 3.1 ± 1.9 points 48 h after *bout* 2 (*P* < 0.05, between groups). Muscle soreness in boys, young adult males and elderly males was 3.1 ± 1.2, 3.2 ± 1.4, and 1.7 ± 0.8 points, respectively, 24 h after *bout* 1 (*P* < 0.05, between elderly males, young adult males, and boys), and 1.7 ± 0.8, 2.1 ± 0.1, and 1.3 ± 0.8 points 48 h after *bout* 2 (*P* < 0.05, between elderly males and young adult males). Differences in muscle soreness 24 h after *bout* 1 and *bout* 2 between boys, young adult and elderly males were 2.4 ± 1.6, 3.1 ± 1.7, and 2.0 ± 0.8 points, respectively (*P* > 0.05 between young adult males and elderly males). Thus, RBE in the CK indicator was significantly greater in young adult males than in boys and elderly males, while in boys it was greater than in elderly males. However, there was no significant difference in RBE of muscle soreness between groups.

## 4. Discussion

The main findings of the present study are as follows: (1) indirect symptoms of muscle damage were more significantly seen in young adult males than in elderly males and boys after the first stretch-shortening exercise bout; (2) RBE was significantly greater in young adult males than in elderly males and boys in the majority of variables; (3) RBE in boys and elderly males was more evident from changes in periphery (muscles); changes were evident in young adults in both, that is, voluntary activation and skeletal muscles.

### 4.1. The Main Causes of Changes in Neuromuscular Function after Eccentric Exercise

The main reasons for a decrease in voluntary and electrically induced muscle performance after SSE (Figure 2) are damage to force-bearing structures [2, 22, 23], changes in the excitation-contraction coupling system [4, 6, 24], and voluntary activation of muscle [19, 25]. The following indirect symptoms of muscle damage manifested within 48 h of SEE: muscle soreness, elevated plasma CK activity, decreased P20, P100, MVC, and *H* of DJs, and increased low-frequency fatigue (Figure 2). The decreases in VA after SSE show that, in our study, central fatigue only occurred in young adult males. Peripheral fatigue was greater than central fatigue, as changes in voluntary and electrically induced torque were greater than changes in VA. SSE decreased P20 to a greater extent than P100, indicating that the muscles were subjected to LFF. LFF is characterized by a relative loss of force at low stimulation frequencies [4, 5, 20, 26].

### 4.2. Why Are the Muscles of Boys and Elderly Males More Resistant to Exercise-Induced Muscle Damage Than Those of Young Adult Males?

The results of our research show that indirect symptoms of exercise-induced muscle damage after SSE are more markedly expressed in young adult males than in boys and elderly males (Figures 2 and 3 and Table 1). This coincides with the results of research done by other scientists indicating that the muscles of adults are less resistant to exercise-induced damage than those of children [8–11]. Since previous research is scarce and has been carried out applying different physical loads and measuring different indirect symptoms in subjects of different age, it is rather difficult to draw generalized conclusions because of age effects on exercise-induced muscle damage. Furthermore, the reasons why the muscles of children are more resistant to exercise-induced muscle damage remain unclear. Soares et al. [9] have shown that 12-year-old children experience less muscle damage (they judged muscle damage on the basis of increase in CK, muscle pain, and decrease in MVCF) than adults, when exercise was performed by elbow extension with the maximal intensity. Webber et al. [8] compared muscle soreness and CK levels in children and adults following a single bout of downhill running (30 min). Although CK levels were elevated 24 h after exercise in both groups, the increase was significantly greater in adults than in children. However, muscle soreness ratings 24 h after exercise were similar in both groups. Duarte et al. [10] drew similar conclusions in their study of markers of muscle overuse in boys, although they made no direct comparison with adults.

One of the possible interpretations why the muscles of adults are less resistant to fatigue when performing SSE is the fact that the muscles of adults contain relatively more fast twitch muscle fibers (type II) than do the muscles of children and elderly males [14–16].

One of the possible interpretations of the fact why the muscles of adults are more sensitive to damage than those of children is that, according to Webber et al. [8], adults, because of their greater body weight, generate more force per fiber unit during eccentric contractions compared with children, thus resulting in greater damage and a greater release of CK into the blood serum. In our study, the average *H* of DJs of young adults, elderly males, and boys was not the same. Therefore, the mechanical stimuli of both young adult and elderly males are also probably not the same.

One can find contradictory data as to resistance of the fast-type and slow-type muscle fibers to eccentric-contraction-induced damage. It has been established, for instance, that when performing eccentric exercise, type IIb muscle fibers seem to be preferentially damaged [27, 28]. Data from research done by other authors indicate that LFF was not different between most of the high-fit and fit subjects [29] and that increasing the muscle oxidative capacity of isometric electrical stimulation training did not protect muscles against eccentric-contraction-induced damage [30]. Skurvydas et al. [18] have shown that muscle fatigue of elite track-and-field sprinters is not greater than that of long-distance runners when repeated SSE is being performed. Therefore, the authors of the present paper have some doubts as to the universal character of the conclusion made by Friden et al. [27] that fast-twitch muscle fibers (type IIb) are more sensitive to exercise-induced damage. Thus, irrespective of the attractive hypothesis that the muscles of children and elderly men are damaged less than those of young adults because their muscles contain fewer type IIb muscle fibers, we cannot assert with conviction that this is the only reason why the muscles of children are less sensitive to exercise-induced damage.

There are no grounds whatever to conclude that the boys and elderly males were not able to fully activate their muscles, for example, type IIb muscle fibers, when performing jumps since they were strongly motivated during SSE (i.e., they were informed of HJ and were asked to perform each jump as high as possible). Research done by other scientists has shown that boys of this age are capable of achieving complete activation of their leg muscles during maximal voluntary contractions [31].

### 4.3. Why Are the Muscles of Elderly Males and Boys More Resistant to LFF Than the Muscles of Young Adult Males?

If the muscles of adult males contain a greater number of type IIb muscle fibers than do those of boys and elderly males, then it is understandable why the muscles of boys and elderly males are more resistant to LFF (Figure 2), since it has been established that in cat skeletal muscles, fatigue-resistant motor units are less susceptible to LFF compared with fast-fatigable motor units [32]. LFF is characterized by a relative loss of force at low frequencies of stimulation, and it is important to mention that the force is not impaired or there is only a relatively low impairment at high frequencies [4, 7, 20, 26]. In our case, there was a decrease in the force evoked not only by low stimulation frequencies (20 Hz), but by high stimulation frequencies (100 Hz) as well (Figure 2).

It has been shown that recovery of force and [Ca^2+^]_*i*_ after fatigue follows a complex time course. Recently, it has been found that the decrease in Ca^2+^ release from sarcoplasmic reticulum associated with fatigue (particularly with LFF) has at least two components: (1) a metabolic component, which recovers within 1 h and (2) a component dependent on the elevation of the [Ca^2+^]_*i*_-time integral, which recovers more slowly [33]. We think that in our study, immediately after the exercise, this metabolic component had an influence on muscle fatigue.

### 4.4. RBE

After *bout* 2, we observed a much less pronounced decrease in voluntary and electrically stimulated torque, as well as lower muscle soreness and plasma CK activity, than after* bout* 1. Our data are in agreement with those of other researchers who have concluded that the adaptation response provides protection against further damage [3, 5]. Different mechanisms have been suggested to explain RBE. The proposed rebuild processes include removal of weakened sarcomeres, longitudinal addition of sarcomeres and strengthening of the cell membrane [24], remodeling of sarcomeres [34], increased connective tissue [35], and/or reorganization of the intermediate filaments [36].

Isometric torque recovery was significantly greater after the second SSE bout in all groups, but this improvement was accompanied by a higher level of voluntary activation only in young adult males (Figure 3). Apparently, a single bout of SSE enlarges the neural drive or prevents central fatigue during a subsequent exercise bout in adult male, at least when full muscle function recovery has been allowed to occur.

A postexercise decrease in central activation was observed in the present study. Thus, SSE-induced muscle damage is associated with a reduction in the adult subjects' ability to voluntarily activate the knee extensor muscles (Figure 2). This agrees with the findings of Prasartwuth et al. [25] that reduced voluntary activation contributes to force loss after eccentric exercises with the elbow flexors. In our study, central fatigue in young adults after *bout* 1 was significantly greater than in boys and elderly males; however, there was no significant difference in VA after *bout* 2. This, however, indicates that only in young adult males, is RBE manifested as well by the change in voluntary activation of exercising muscle.

## 5. Conclusions

In conclusion, (1) indirect symptoms of muscle damage (muscle soreness, CK levels, and prolonged impairment of muscle function during both voluntary and electrically stimulated contractions at low (10–20 Hz) and high frequencies (100 Hz)) were more significantly evident in young adult males than in elderly males and boys after the first stretch-shortening exercise bout; (2) RBE was more pronounced in young adult males than in boys and elderly males.

## Figures and Tables

**Table 1:** Creatine kinase activity (CK) changes after *bout* 1 and *bout* 2 in boys, young adult males, and elderly males (mean ± SD).

CK, IU/L	Before	24 h after exercise	48 h after exercise
Boys			
*bout * 1	78.8 ± 50.7	422.5 ± 211.5^∗#^	311.8 ± 236.2^∗#^
*bout* 2	75.5 ± 46.1	132.8 ± 68.2*	102.8 ± 55.4
Young adult			
*bout* 1	106.0 ± 38.9	1102.2 ± 640.9^∗#$^	1090.2 ± 784.7^∗#$^
*bout* 2	90.4 ± 40.8	401.9 ± 182.5^∗$^	194.2 ± 76.7*
Elderly			
*bout* 1	82.7 ± 24.2	224.6 ± 113.6*	340.8 ± 149.8^∗#^
*bout* 2	92.5 ± 47.5	164.7 ± 81.5*	144.5 ± 55.3*

*P < 0.05, compared with before; ^#^ *P* < 0.05, compared with *bout* 1; ^$^ *P* < 0.05, compared with elderly males and boys.
